# Alterations in EGFR and Related Genes following Neo-Adjuvant Chemotherapy in Chinese Patients with Non-Small Cell Lung Cancer

**DOI:** 10.1371/journal.pone.0051021

**Published:** 2013-03-08

**Authors:** Shuhang Wang, Tongtong An, Jianchun Duan, Lijian Zhang, Meina Wu, Qinghua Zhou, Jinfeng Chen, Minglei Zhuo, Lu Yang, Yuyan Wang, Hua Bai, Jie Wang

**Affiliations:** 1 Key Laboratory of Carcinogenesis and Translational Research (Ministry of Education), Department of Thoracic Medical Oncology, Peking University School of Oncology, Beijing Cancer Hospital and Institute, Beijing, China; 2 Key Laboratory of Carcinogenesis and Translational Research (Ministry of Education), Department of Thoracic Surgery, Peking University School of Oncology, Beijing Cancer Hospital and Institute, Beijing, China; 3 Department of Thoracic Surgery, Tianjin Medical University General Hospital, Tianjin, China; Univesity of Texas Southwestern Medical Center at Dallas, United States of America

## Abstract

**Introduction:**

Genetic aberrancies within epidermal growth factor receptor (EGFR) pathway are associated with therapeutic outcomes of EGFR-tyrosine kinase inhibitors (TKIs) in advanced non-small cell lung cancer (NSCLC). However, the impact of chemotherapy on EGFR-related genes alterations has not been defined in NSCLC. Our study aims to investigate the impact of neoadjuvant chemotherapy (Neoadj-Chemo) on EGFR activating mutations and associated EGFR-TKIs resistance-related genes.

**Patients and Methods:**

Matched tumor samples were obtained retrospectively from 66 NSCLC patients (stages IIb–IIIb) corresponding to pre- and post- Neoadj-Chemo. EGFR mutations were detected by denaturing high performance liquid chromatography (DHPLC) and confirmed by Amplification Refractory Mutation System technology (ARMS), KRAS mutations, T790M mutation and c-MET amplification were identified using Polymerase Chain Reaction-Restriction Fragment Length Polymorphism (PCR-RFLP), ARMS, and real-time PCR, respectively.

**Results:**

Before Neoadj-Chemo, EGFR mutations were identified in 33.3% (22/66) of NSCLC patients. Only 18.2% (12/66) of patients carried EGFR mutations after Neoadj-Chemo (p = 0.013). The median peak value of EGFR 19 exon mutations decreased non-significantly after Neoadj-Chemo. KRAS mutation rate decreased from 4.6% (3/66) to 3.0% (2/66) with Neoadj-Chemo. Although the overall percentage of patients exhibiting c-MET amplifications (6.1% [4/66]) did not change with Neoadj-Chemo, two patients transitioned from negative to positive c-MET amplification, and two patients reversed these changes post-Neoadj-Chemo. T790M mutations were absent from all samples.

**Conclusion:**

Neoadjuvant chemotherapy tends to decrease the mutation frequency of EGFR mutation and downstream genes, which suggests that real-time samples analysis for genetic aberrancies within EGFR pathways have important value to delineate specific patient populations and facilitate individualized treatment.

## Introduction

Epidermal growth factor receptor-tyrosine kinase inhibitors (EGFR-TKIs) represent revolutionary personalized therapies for NSCLC patient, a subset of who carry specific EGFR mutations that are predictive of a favorable clinical response to EGFR-TKIs [1]–[3]. Somatic mutations in exons 19 or 21 constitute about 90% of all EGFR-activating mutations, and the identification of these mutations can be applied to the choice of lung cancer therapy. Several phase III clinical trials have confirmed that the presence of EGFR-activating mutations is predictive of a favorable outcome with EGFR-TKIs (i.e., gefitinib and erlotinib), compared with doublet chemotherapy, as first-line therapy for NSCLC, and in both Asian and Caucasian advanced NSCLC patients [4]–[7]. All above suggest that EGFR mutation status governs the outcome to EGFR-TKIs, regardless of ethnicity [8].

The outcome of EGFR-TKI therapy is determined not only by the presence of EGFR sensitizing mutations, but also by EGFR resistant or its bypass or downstream related genes aberrances. Specifically, EGFR T790M mutation, was identified mechanism of both acquired and primary EGFR-TKI resistance [9], and amplification of the c-MET oncogene [10] are described as acquired resistance, whereas KRAS mutation is associated with primary resistance [11]. These findings have led to clinical trials applying novel therapies targeted to the resistance mechanisms as well as promising preliminary results in laboratory and clinical studies.

The detection of EGFR mutations currently is recommended for the selection of patients who could benefit from first-line EGFR-TKI therapy. However, it is unknown whether the status of EGFR mutation and downstream resistance-related genes aberrances (*i.e.*, KRAS mutations, T790M, or c-MET amplification) are consistent in pre- and post-chemotherapy samples. Therefore, it is necessary to evaluate the impact of chemotherapy on tumor molecular profiles. Chin et al reported that prior exposure to platinum agents may reduce the benefit from subsequent treatment with EGFR-TKI for an erlotinib-sensitive EGFR-mutant NSCLC cell via the phosphatidylinositol 3-kinase/AKT survival pathway [12]. Our recent study investigated influence of chemotherapy on EGFR mutated frequencies using two cohorts including plasma DNA in advanced NSCLC and pre- and post-operative tissue samples in patients with locally advanced NSCLC, and explored the possible mechanism of chemotherapy related alteration of EGFR mutation. The results suggested that chemotherapy may reduce E*GFR* mutation frequencies in patients with NSCLC, a likely result of a preferential response of sub-clones with EGFR mutations in tumors with heterogeneous tumor cell populations [13]. To our knowledge, few studies have evaluated clinical samples for the influence of chemotherapy on the EGFR-TKI resistance related genes.

Therefore, we hypothesized that chemotherapy might influence the mutation frequency of EGFR mutation and downstream genes, thus it might also cause impact on the role of these genes working as selective markers in individualized treatment of EGFR-TKI. As a continuity of our prophase study, the current study explored the impact of chemotherapy on both EGFR activating mutations,especially, assessed variations in mutation quantity in EGFR exon 19 and clinical significance, and further investigated potential alterations of EGFR-TKI resistance-related genes, such as T790M, KRAS and c-MET aberrances using the same cohort of matched tumor tissue samples of pre- and post- Neoadj-Chemo from stage IIb-IIIb NSCLC patients.

## Patients and Methods

### Patient

Patients enrolled in this retrospective study were older than 18 years and exhibited stage IIB-IIIB NSCLC with dimensionally measurable disease before surgery. Eligible patients also had an ECOG (Eastern Cooperative Oncology Group) performance status of 0-2 and had received 2-4 cycles of Neoadj-Chemo without any previous chemotherapy or biologic/immunologic treatment. All patients provided matched tissue samples from biopsies performed pre- Neoadj-Chemo and from resections post-Neoadj-Chemo.

Sixty-six patients who were screened from the our database established in 1999, including more than 1,900 patients with clinical data met the above criteria and were treated in Beijing Cancer Hospital from September 2001 to June 2010 (according to the prerequisite of the enrollment this is a 9 years span, but most of the samples were collected from 2005 to 2010). The available chemotherapy regimens involved platinum-based drugs (*e.g.*, cisplatin or carboplatin in combination with gemcitabine or taxanes). The radiographic response to Neoadj-Chemo was determined using the RECIST (Response Evaluation Criteria in Solid Tumors) guidelines [14]. All patients provided written informed consent for biomarker analysis in their first-time hospitalization. The study protocol was approved by the Institutional Ethics Committee of Beijing Cancer Hospital.

### Ethics Statement

All patients provided written informed consent for biomarker analysis. The study protocol was approved by The Institutional Ethics Committee at Beijing Cancer Hospital. We did not conduct research outside our country of residence. All participants provide their written informed consent to participate in this study. Our ethics committees approved this consent procedure. The individual in this manuscript has given written informed consent to publish these case details.

### Specimen collection and DNA extraction

Tumor tissues obtained before and after Neoadj-Chemo treatments were processed for genomic DNA isolation using E.Z.N.A FFPE DNA kits (Omega Biotek, USA). To avoid the influence of Neoadj-Chemo-induced necrosis of tissues on the EGFR and related genes aberrance detections, post-operative samples were macro-dissected from paraffin-embedded surgical tissue sections to ensure that only tumor tissues were obtained. Tumor contents were recorded for each sample using immediately adjacent tissue sections.

EGFR mutation detection by denaturing high performance liquid chromatography (DHPLC) and Amplification Refractory Mutation System (ARMS)

We analyzed all matched samples in the same condition in order to equalize the detection conditions. The EGFR exon 19 deletion or exon 21 substituted mutations were detected according to the method reported by us previously [15].

ARMS, a more sensitive method, was used to re-evaluate the cases with EGFR mutation discrepancies pre- and post- neoadjuvant chemotherapy [13].

A semi-quantitative analysis of exon 19 mutation abundance was performed by calculating the ratio between the peak heights of mutant (M) and wild-type/normal (W) products (i.e., M/W). This analysis was not extended to exon 21 because the M and W peaks overlapped in this exon.

### PCR-RFLP for KRAS mutations

PCR-RFLP was performed to analyze KRAS mutation status according to standard protocols [16]. PCR primers were designed to amplify sequences surrounding codons 12 and 13 in exon 1 of KRAS; these codons are involved in approximately 85% of activating KRAS mutations.

### Quantitative real-time PCR for c-MET

Paired tumor samples were analyzed for c-MET gene copy number using real-time PCR detection according to standard protocols. A tumor sample was defined as amplification-positive if its ratio value exceeded the following: mean (M)+2×standard deviation (SD) [17]. HCC827 (Homo sapiens (human) lung adenocarcinoma, ATCC number CRL-2868) was used as negative control.

### Amplification Refractory Mutation System detection of EGFR T790M

Amplification Refractory Mutation System (ARMS) technology was used to detect the T790M mutation. The detection method and discrimination criteria were according to manufactory (Amoy Diagnostics Co., LTD).

### Statistical analysis

Frequency tabulation and summary statistics were provided to characterize the data distribution. McNemar's test was applied to compare the change of mutation status (*i.e.*, EGFR [including T790M] mutations, KRAS mutation, and c-MET amplification) before and after treatment. Cochran-Armitage trend test was used to test whether the change in mutation status was associated with clinical outcome in terms of partial response, stable disease. The associations of unpaired categorical variables were analyzed using the chi-square test, except that Fisher's exact test was used for small sample sizes (n<5 in any cell of the contingency table). Wilcoxon rank sum test was applied to compare the mutant abundance of EGFR 19 exon between pre- and post Neoadj-chem. Statistical significance was set at a level of 0.05. Two-sided tests were performed in all settings. All calculations were performed using SAS Version 10.0 (SAS Institute, Inc., Cary, NC).

## Results

### Patient characteristics and EGFR mutation status

The baseline characteristics of the study population are presented in Table 1. The 66 patients included 50 males and 16 females with an age range of 35-78 years (median 60 years). The patients included 35 (53.0%) cases of squamous cell carcinoma, 27 (40.9%) cases of adenocarcinoma, and 4 cases of adenosquamous carcinoma. All patients received Neoadj-Chemo; 63 (95.5%) were treated with platinum plus paclitaxel (median 2 cycles; range 2–4 cycles). Based on the 2009 American Joint Committee on Cancer's lung cancer tumor-node-metastasis staging system, 9 patients were diagnosed as stage IIb, 35 as stage IIIa, and 22 as stage IIIb before surgery; and after surgery, 11 as _p_stage IIa, 27 as _p_stageIIb, 23 as _p_stage IIIa, 5 as _p_stage IIIb. With Neoadj-Chemo treatment, 39 of 66 patients achieved a partial response (PR), 27 patients were classified as stable disease (SD), and none exhibited progressive disease (PD).

**Table 1:** Gene mutation status of baseline according to Clinical Characteristics.

Characteristic	Patient	EGFR mut+	CMET amp+	KRAS mut+
	N = 66	N = 22	N = 4	N = 3
Age				
≤60 years	30(45.5)	10(45.5)	0	1(33.3)
>60 years	36(54.5)	12(54.5)	4(100.0)	2(66.7)
Sex				
Male	50(75.8)	16(72.7)	4(100.0)	3(100.0)
Female	16(24.2)	6(27.3)	0	0
Histologic subtype				
Adenocarcinoma	27(40.9)	13(59.1)	3(75.0)	0
Squamous carcinoma	35 (53.0)	8(36.4)	1(25.0)	3(100.0)
adenosquamous carcinoma	4 (6.1)	1(4.5)	0	0
Smoking status				
Former smoker	32(48.5)	8(36.4)	2(50.0)	2(66.7)
Never smoker	34(51.5)	14(63.6)	2(50.0)	1(33.3)
Stage(pre-operation)				
IIB	9(13.6)	4(18.2)	0	0
IIIA	35(53.1)	15(68.2)	2(50.0)	1(66.7)
IIIB	22(33.3)	3(13.6)	2(50.0)	2(33.3)
Chemotherapy				
PR	39(59.1)	13(59.1)	4(100.0)	1(33.3)
SD	27(40.9)	9(40.9)	0	2(66.7)

Abbreviation: mut+−mutation positive, amp+−amplification positive

### EGFR mutation variation pre- and post-Neoadj-Chemo

**Figure 1:**
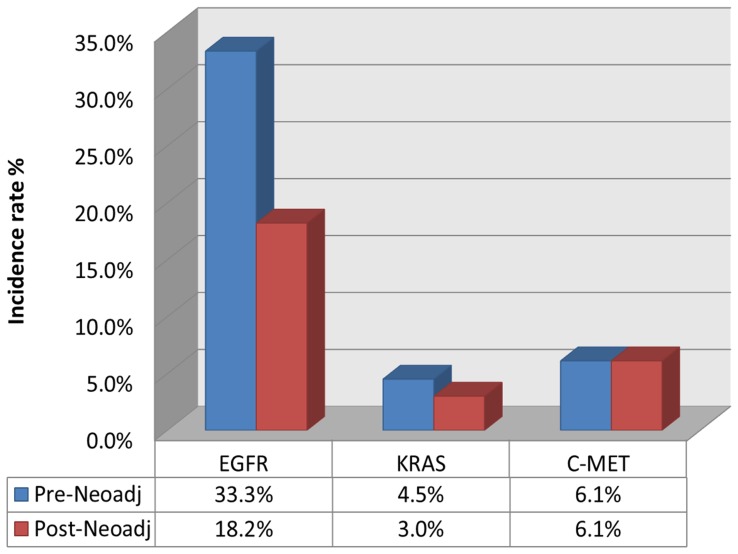
Variation pre- and pro- Neoadj-Chemo. Neoadj-Chemo: Neoadjuvant chemotherapy; EGFR: EGFR mutation; KRAS: KRAS mutation; C-MET: C-MET amplification

Among 66 NSCLC patients, the pre-Neoadj-Chemo EGFR mutation rate was 33.3% (22/66), including 12 patients carrying mutations in exon 19, 9 patients with mutations in exon 21, and 1 patient with mutations in both exons. Post-Neoadj-Chemo, the EGFR mutation rate decreased to 18.2% (12/66), including 7 patients with mutations in 19 exon and 5 with mutations in exon 21 (McNemar's test P = 0.013; Figure 1). The consistency of EGFR mutations in pre- and post-chemotherapy specimens was 78.8% (52/66). With Neoadj-Chemo treatment, the EGFR status transitioned from mutant to wild-type in 12 patients; the opposite occurred in 2 patients.

In pre-Neoadj-Chemo samples, the EGFR mutation rate was 55.6% (15/27) in adenocarcinoma, which is higher than measured in patients with squamous cell carcinoma (17.1% [6/35]). However, the EGFR mutation rates in the patients who underwent Neoadj-Chemo dropped to 33.3% (9/27) in adenocarcinoma and 8.57% (3/35) in squamous cell carcinoma, with a non-significant difference (P>0.05). The four patients with adenosquamous carcinoma composed too small of a group for statistical analysis (Table 1).

### Ratio of DHPLC peak heights associated with EGFR mutation in exon 19

**Figure 2:**
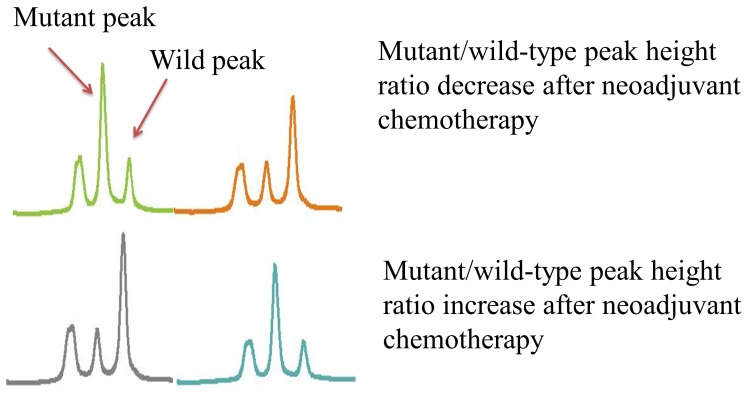
Ratio of mutant/wild-type peak height change after neo-adjuvant chemotherapy.

To assess variations in mutation quantity, mutations in EGFR exon 19 were semi-quantitatively detected from 13 patients carrying mutations in this exon (Figure 2). The ratio of peak heights between pre- and post-Neoadj-Chemo changed in all patients. Following Neoadj-Chemo, exon 19 mutations transitioned from positive to negative in 7 patients. The peak heights exhibited a sharp decrease in three patients, whereas they rose in three other patients. The median M/W ratio pre-Neoadj-Chemo was 0.50; the ratio fell to 0.24 post-Neoadj-Chemo, a non-significant difference (P = 0.078).

### EGFR mutation variation and efficacy of Neoadj-Chemo

All patients were evaluated for their response after Neoadj-Chemo. 39 patients (59.1%) achieved PR, 27 patients (40.9%) reached SD, and none exhibited PD (Table 1). Among the 66 patients, 12 patients' EGFR status changed from mutation to wild-type after treatment, 2 cases had reverse shift, 52 remained wild type (n = 42) or mutation (n = 10) status pre- and post- neoadj-Chemo. The PR rates in these subgroups were 58.33% (7/12), 50% (1/2), and 59.6% (31/52), respectively. The clinical response was not significantly associated with the change of EGFR mutation status (P = 0.13, Cochran-Armitage trend test).

### KRAS mutation variation pre- and post-Neoadj-Chemo

The KRAS mutation rate varied from 4.5% (3/66) pre-Neoadj-Chemo to 3.0% (2/66) after treatment (Table 2). The consistency of KRAS mutations pre- and post-chemotherapy was 95.5% (63/66). Two patients retained their KRAS mutation statuses after treatment, whereas one patient with KRAS mutation transitioned to wild status after post-Neoadj-Chemo.

**Table 2:** Gene mutation variation after neo-adjuvant chemotherapy in each case.

case	Response	Sex	Age	Histology	Smoke status	stage	EGFR		CMET		KRAS	
							Pre-Post-		Pre-Post		Pre-Post-	
1	PR	male	70	Squ	Yes	IIIB	W	W	N	N	W	W
2	PR	male	50	Ade	Yes	IIB	W	M	N	N	W	W
3	PR	male	69	Squ	No	IIIA	W	W	N	N	W	W
4	PR	male	58	Squ	Yes	IIIB	M	W	N	N	W	W
5	PR	male	69	Ade	Yes	IIIA	M	M	A	N	W	W
6	PR	male	60	Ade	Yes	IIIA	M	M	N	N	W	W
7	PR	male	64	Squ	No	IIB	M	W	N	N	W	W
8	PR	female	56	Ade	No	IIIA	W	W	N	N	W	W
9	PR	male	76	Squ	Yes	IIB	W	W	N	N	W	W
10	PR	male	47	Ade	Yes	IIIB	W	W	N	N	W	W
11	PR	female	59	AdeSqu	No	IIIA	W	W	N	N	W	W
12	PR	male	55	Squ	Yes	IIIA	W	W	N	N	W	W
13	PR	male	68	Squ	No	IIIB	W	W	N	N	W	W
14	PR	male	70	Squ	Yes	IIIB	W	W	N	N	M	W
15	PR	male	63	Squ	Yes	IIIB	W	W	N	N	W	W
16	PR	female	63	Squ	No	IIIA	W	W	N	N	W	W
17	PR	female	75	Squ	No	IIIA	W	W	N	N	W	W
18	PR	male	47	Squ	Yes	IIIA	W	W	N	N	W	W
19	PR	male	72	Ade	Yes	IIB	W	W	N	N	W	W
20	PR	female	70	Squ	No	IIIB	W	W	N	N	W	W
21	PR	male	69	Ade	No	IIIA	W	W	A	A	W	W
22	PR	male	68	Squ	Yes	IIIB	W	W	A	A	W	W
23	PR	female	52	Ade	No	IIIA	M	W	N	N	W	W
24	PR	male	40	Squ	No	IIIA	M	W	N	N	W	W
25	PR	male	60	Ade	No	IIIA	M	M	N	N	W	W
26	PR	male	69	Squ	Yes	IIIA	M	M	N	N	W	W
27	PR	male	59	AdeSqu	Yes	IIIA	M	W	N	N	W	W
28	PR	male	65	Squ	No	IIIA	M	W	N	N	W	W
29	PR	male	76	Squ	No	IIIA	W	W	N	N	W	W
30	PR	male	55	Squ	Yes	IIIB	W	W	N	N	W	W
31	PR	female	65	Ade	No	IIIB	W	W	N	N	W	W
32	PR	female	70	Squ	No	IIIA	W	W	N	N	W	W
33	PR	male	43	Ade	Yes	IIIB	W	W	N	N	W	W
34	PR	male	59	Squ	Yes	IIIB	W	W	N	N	W	W
35	PR	male	56	Squ	Yes	IIIB	W	W	N	N	W	W
36	PR	male	64	Ade	No	IIIB	W	W	A	N	W	W
37	PR	male	68	Ade	No	IIIB	M	M	N	N	W	W
38	PR	male	76	Squ	Yes	IIIA	M	M	N	N	W	W
39	PR	male	66	Ade	No	IIIA	M	W	N	N	W	W
40	SD	male	57	Squ	Yes	IIB	W	W	N	N	W	W
41	SD	female	62	Ade	No	IIB	M	M	N	N	W	W
42	SD	male	53	Squ	No	IIB	W	M	N	N	W	W
43	SD	female	61	Ade	No	IIIA	W	W	N	N	W	W
44	SD	male	62	Ade	Yes	IIIA	W	W	N	N	W	W
45	SD	male	59	Squ	No	IIIA	W	W	N	N	M	W
46	SD	male	38	AdeSqu	Yes	IIIA	W	W	N	N	W	W
47	SD	male	57	Ade	Yes	IIIA	W	W	N	N	W	W
48	SD	male	78	Squ	No	IIIA	W	W	N	N	W	W
49	SD	male	75	Squ	No	IIIA	W	W	N	N	W	W
50	SD	female	48	Ade	No	IIIA	W	W	N	N	W	W
51	SD	male	45	Squ	Yes	IIIA	W	W	N	A	W	M
52	SD	female	73	Ade	No	IIB	M	M	N	N	W	W
53	SD	female	66	Ade	No	IIB	M	M	N	N	W	W
54	SD	male	58	Ade	Yes	IIIA	M	W	N	N	W	W
55	SD	female	48	Ade	No	IIIA	M	W	N	N	W	W
56	SD	male	49	Squ	No	IIIA	M	W	N	A	W	W
57	SD	female	57	Ade	No	IIIB	M	W	N	N	W	W
58	SD	female	46	Ade	No	IIIA	W	W	N	N	W	W
59	SD	male	72	Squ	Yes	IIIB	W	W	N	N	M	M
60	SD	male	64	Squ	Yes	IIIB	W	W	N	N	W	W
61	SD	male	54	Ade	Yes	IIIB	W	W	N	N	W	W
62	SD	male	71	Squ	No	IIIB	W	W	N	N	W	W
63	SD	male	68	Squ	Yes	IIIB	W	W	N	N	W	W
64	SD	male	51	AdeSqu	Yes	IIIB	W	W	N	N	W	W
65	SD	male	58	Ade	Yes	IIIA	M	M	N	N	W	W
66	SD	male	63	Squ	No	IIIA	M	W	N	N	W	W

Abbreviation: Ade-adenocarcinoma, Squ-squamous carcinoma, AdeSqu-adenosquamous carcinoma; PR-partial response, SD- stable disease; pre-: pre-neoadjuvant chemotherapy, post-: post-neoadjvant chemotherapy; W: wild type; M: mutant type; N: negative; A: amplification. For smoke status, Yes: smoker; No: non-smoker.

### c-MET amplification pre- and post-Neoadj-Chemo

The overall c-MET amplification ratio did not change with Neoadj-Chemo treatment (6.0% [4/66]) (Table 2). However, 4 of 66 patients exhibited a shift in c-MET amplification status. Specifically, two cases transitioned from amplification-negative to -positive, and two exhibited the reverse transition. Two patients exhibiting c-MET amplification pre-treatment retained their amplification status after treatment. The consistency of c-MET amplification pre- and post-Neoadj-Chemo was 93.9% (62/66). Interestingly, two patients whose c-MET amplification status changed from positive to negative obtained PR with Neoadj-Chemo. The two patients with c-MET changes from negative to positive exhibited SD with Neoadj-Chemo.

### T790M mutation status pre- and post-Neoadj-Chemo

T790M mutations were absent from all samples, as determined using ARMS technology.

## Discussion

Previous studies have not evaluated whether the molecular profiles of tumor tissues change between the initial diagnosis and post-chemotherapy. Based on our prophase study results [13], we further demonstrated that Neoadj-Chemo affects not only the EGFR mutation status, but also the aberrant mutation status of EGFR related downstream or bypass genes in NSCLC patients. These results support chemotherapy-related molecular heterogeneity.

EGFR mutations are well-established predictors of the outcome to first-line EGFR-TKI therapy [4]–[7]. However, it has been unclear whether EGFR mutation status detected using initially diagnostic samples also accurately predicts second- or third-line EGFR-TKI therapies. At present, the majority of Chinese patients with advanced NSCLC undergo EGFR-TKI only as second- or third-line therapies, potentially owing to medical insurance policies. Therefore, it is important to know the impact of first-line chemotherapy on EGFR mutation status to guide the selection of second-line EGFR-TKI therapy. A high incidence of alterations in EGFR mutation status with chemotherapy would warrant a second biopsy prior to initiating second- or third-line EGFR-TKI therapy.

In our study, the overall EGFR mutation rate significantly decreased in post-Neoadj-Chemo tissue samples compared with pre-Neoadj-Chemo ones in patients with early stage NSCLC. We detected a discordant rate of 18.2% (12/66), which included patients who transitioned from EGFR mutation-positive to -negative (n = 10) and the reverse (n = 2). No significant differences have been identified regarding the incidence of EGFR mutations with early-to-advanced staging of NSCLC [18]. We therefore presumed that Neoadj-Chemo-related alterations in EGFR mutation observed in early-stage NSCLC might be analogous to changes induced by first-line chemotherapy in advanced NSCLC cases. Our results suggest that the EGFR mutation status assessed prior to chemotherapy may not accurately reflect the mutation status post-chemotherapy, which supports a “real-time” tumor profiling in the personalized therapy of NSCLC.

The mechanisms contributing to chemotherapy-related shifts in EGFR mutation status remain unclear. Heterogeneous intra-tumoral EGFR mutations and different sensitivities of EGFR mutant and wild-type tumor cells to chemotherapy may be associated with alterations in overall EGFR mutation status following chemotherapy [4], [19]. Yatabe et al [20] suggested that, in lung adenocarcinomas, heterogeneous distributions of EGFR mutations across cells are extremely rare. However, several other studies have observed heterogeneity in EGFR gene expression, mutation or amplification between primary and metastatic tumors or among intra-tumoral foci, with different discordance rate [21]–[23]. Okami et al [24] reported that patients whose tumors included both EGFR-mutated and -wild cells had significantly shorter progression-free survival lengths following gefitinib therapy compared with patients whose tumors consisted of EGFR-mutated cells only. Zhou et al [25] showed patients with a high introtumoral abundance of EGFR mutations may benefit more than those with low abundance of EGFR mutation. All these findings suggest that the intratumoral heterogeneity of EGFR mutational status may impact the outcome of EGFR-TKI therapy.

Our previous study detected variations in EGFR mutation status through microdissection of different foci from 85 advanced NSCLC patients following palliative surgery resection. The results showed that approximately 30% of specimens contained both EGFR mutant and wild-type cells with the proportion of EGFR mutant cells ranging from 1% to 90% [13]. Multiple pre-clinical and clinical trials indicated that EGFR mutants were more sensitive not only to EGFR-TKIs but also to chemotherapy, as compared with those carrying wild-type EGFR [4], [21], [26]. We speculate that chemotherapy may selectively kill and inhibit mutant clones within the tumor, whereas the clones exhibiting wild-type EGFR may proliferate abundantly following chemotherapy. Such a phenomenon could explain why EGFR mutation status shifted from EGFR-mutant to wild-type following chemotherapy. Whereas very low frequent mutation cells (1%∼5%) were found only in the microdissected samples [13], which may be associated with variation of EGFR from wild-type to mutation after chemotherapy. These small clones of EGFR mutation may be selected and proliferated after exposed to chemotherapy.

The discordance of EGFR mutation status between biopsy and surgically resected samples pre- and post-Neoadj-Chemo, respectively, also may be attributed to sampling differences resulting from intra-tumoral heterogeneity. Specifically, small biopsy samples with limited materials might not represent the complete biological features of the tumor, and miniscule proportions of mutant cells may be overlooked. However, the current study observed a higher incidence of EGFR-related gene aberrances in biopsy samples of pre-Neoadj-Chemo compared with surgery resected samples of post-Neoadj-Chemo. This suggests that the discordance of EGFR mutation status observed in the samples of pre- and post-Neoadj-Chemo didn't derive from sampling bias.

We demonstrated that Neoadj-Chemo treatment of NSCLC patients impacted not only EGFR mutation status but also aberrancies in related downstream genes, including KRAS and c-MET. The KRAS mutation rate was 4.6% (3/66) pre-Neoadj-Chemo and decreased to 3.0% (2/66) following therapy. Although the c-MET amplification ratio pre- and post-Neoadj-Chemo did not change (6.1% [4/66]), 4 patients exhibited shifts. Specifically, two cases transitioned from amplification-negative to -positive with Neoadj-Chemo, and two patients exhibited the reverse change. Our results suggest that c-MET and KRAS gene aberrancies exist in untreated tissue samples at low frequencies; this is consistent with studies by Turke et al [27] and Maheswaran et al [28]. Together with observed EGFR mutation alterations, these results suggest that repeating tumor biopsies during the course of a patient's disease may better guide the choice of therapeutic regimen.

We cannot fully explain the potential mechanisms contributing to alterations in these resistance-related genes during the course of chemotherapy. Several studies have recently reported similar alterations in EGFR resistant genes [27]–[30]. Sequist et al [29] performed a longitudinal analysis of genetic and phenotypic changes in 37 patients with erlotinib-resistant NSCLCs carrying EGFR mutations. Using serial biopsies, the authors reported that T790M and PIK3CA (phosphoinositide-3-kinase catalytic, alpha polypeptide) mutation were lost in the absence of continued selective pressure from EGFR inhibitors. Such cancers may become sensitive to a second round of treatment with EGFR inhibitors. To our knowledge, this study first utilized repeat biopsies to examine dynamically the change in genotype and phenotype with EGFR-TKI and chemotherapy. Chin et al reported that prior exposure to platinum agents could affect the subsequent response to erlotinib in a cell culture model of an erlotinib-sensitive EGFR-mutant NSCLC cell line [12]. This occurs via a persistently activated PI3K/AKT pathway and is facilitated by a cisplatin-induced reduction in PTEN (phosphatase and tensin homolog)function. The mechanisms involved in this chemotherapy-related change may be profound, and epigenetics may play an important role. This warrants further investigation.

We found that EGFR T790M mutation was absent in both pre- and post-Neoadj-Chemo samples. It's inconsistent with the findings of Maheswaran et al [28] and Rosell et al [31] who reported that the T790M mutation could be detected in pre-treated tumor-biopsy specimens. Two hypotheses currently exist to explain the mechanism of T790M mutation. The acquired resistance hypothesis purports that exposing to EGFR-TKIs induces a second point mutation, resulting in a threonine-to-methionine change at position 790 of EGFR. Alternatively, the selective resistance hypothesis suggests that the T790M mutation might exist in patients as small clones prior to treatment; these resistant clones may proliferate after exposed to gefitinib or erlotinib. However, in our study, the absence of T790M mutation in both pre- and post- Neoadj-Chemo samples has been confirmed independently by Amoy Diagnostics Co., LTD. Possible reason for the inconsistency of T790M status reported by us and Maheswaran et al [28] may be related with ethnic difference in certain molecular profiles, which need to be validated in further studies.

Limitations were that: this study is a small samples and retrospective study and the patients with squamous cell carcinoma (SQC) in this study accounted for 53%. Former study showed the mutation rate of SQC in Caucasians is no more than 3.6% [32]. However in Chinese advanced NSCLC population, mutation rate of SQC patients was much higher than that of Caucasian patients (12–20%) [33]–[34], which was consistent with results in our center (17.8%) [35]. In current study, the mutation rate of squamous cell carcinoma 23.5%, this is right around the level of Chinese population.

## Conclusion

We evaluated variations in EGFR mutations (including T790M), KRAS mutations, and c-MET amplification both pre- and post- Neoadj-Chemo treatment. Our findings supported that chemotherapy could affect molecular biomarker profiles and indirectly indicate the presence of tumor heterogeneity. Therefore, the effects of treatments on tumor profiling should be evaluated before making decisions regarding second- or third-line EGFR-TKI therapies for NSCLC patients. The preparation of real-time molecular biomarker profiles for EGFR-TKIs is suggested to delineate specific patient populations and facilitate individualized treatment.
